# Pulmonary *Mycobacterium kansasii* Infection Mimicking Malignancy on the ^18^F-FDG PET Scan in a Patient Receiving Etanercept: A Case Report and Literature Review

**DOI:** 10.1155/2014/973573

**Published:** 2014-10-20

**Authors:** Zaw Min, Mohan Amlani

**Affiliations:** ^1^Core Faculty, Division of Infectious Diseases, Allegheny General Hospital, Allegheny Health Network, 420 East North Avenue, East Wing, Suite 407, Pittsburgh, PA 15212, USA; ^2^Baltimore Washington Medical Center, 8021 Ritchie Highway, Pasadena, MD 21122, USA

## Abstract

A 66-year-old male presented with chest pain, malaise, generalized weakness, and weight loss. He had been receiving etanercept injection for rheumatoid arthritis. Chest X-ray revealed a right upper lobe mass. Chest computed tomography (CT) showed a right apical mass, highly suggestive of a Pancoast tumor. The thoracic fluorine-18 fluoro-deoxy-glucose (^18^F-FDG) positron emission tomography (PET) scan demonstrated significantly high metabolic pulmonary lesions with the standardized uptake value (SUV) of 12.5, consistent with lung cancer. The patient underwent bronchoscopy and bronchoalveolar lavage (BAL). BAL cytology was negative for malignant cells. BAL acid fast bacilli (AFB) smears were positive, and *Mycobacterium kansasii* was eventually isolated. He received a 12-month course of rifampin, isoniazid, and ethambutol. Interval resolution of pulmonary lesions was noted on follow-up serial CT chest studies. There has been increasing incidence of nontuberculous mycobacterial infections reported in patients treated with the antitumor necrosis factor-alpha (anti-TNF-alpha) agents. Infectious foci have an increased glucose metabolism which potentially causes a high FDG uptake on the ^18^F-FDG PET scan, leading to undue anxiety and cost to the patients. This is the first reported case of pulmonary *M. kansasii* infection with a positive thoracic ^18^F-FDG PET study mimicking malignancy in a patient on etanercept.

## 1. Introduction

Tumor necrosis factor-alpha (TNF-alpha) inhibitors have been used in a wide range of inflammatory diseases (e.g., rheumatoid arthritis, psoriasis, and inflammatory bowel disease). TNF-alpha plays an integral role in formation of granulomas to contain mycobacterial or fungal infections [1]. Patients receiving anti-TNF agents have a significant risk of acquiring granulomatous tuberculous or nontuberculous mycobacterial infections [2, 3]. Infected granulomas are generally composed of highly metabolic inflammatory cells, including histiocytes and macrophages, which could result in a false positive fluorine-18 fluoro-deoxy-glucose (^18^F-FDG) positron emission tomography (PET) scan [4]. Herein, we described the first case report of a patient on etanercept presenting with pulmonary* Mycobacterium kansasii* infection with a highly positive ^18^F-FDG PET scan imaging study that was initially mistaken as primary lung cancer.

## 2. Case Description

**Figure 1:**
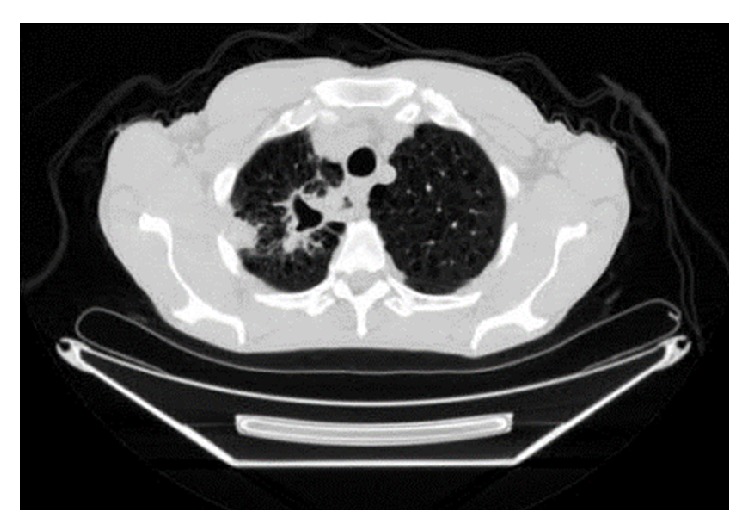
Axial view of CT chest showed a peripheral consolidation (1.5 × 2 × 1 cm) and an apical cavitary lesion (2.5 × 3 × 3.5 cm) in the right upper lobe.

**Figure 2:**
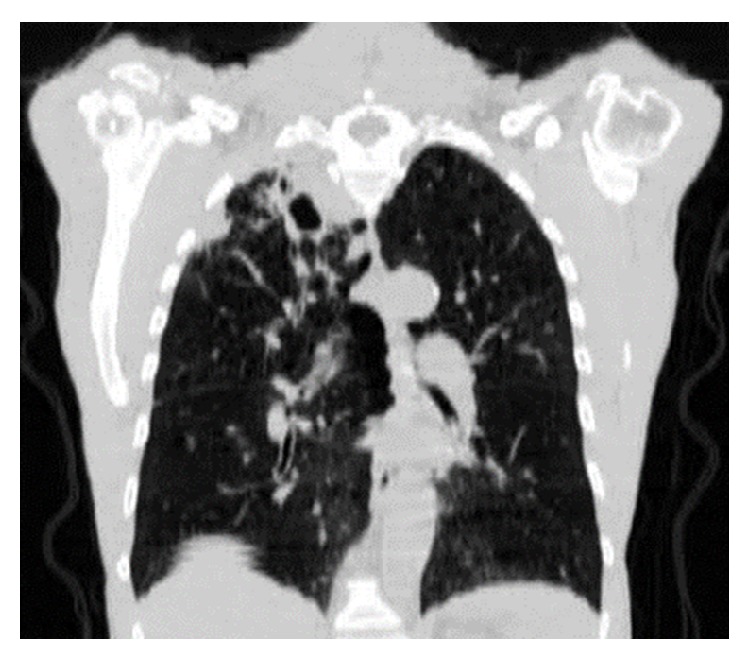
Coronal view of CT chest showed a peripheral consolidation (1.5 × 2 × 1 cm) and an apical cavitary lesion (2.5 × 3 × 3.5 cm) in the right upper lobe.

**Figure 3:**
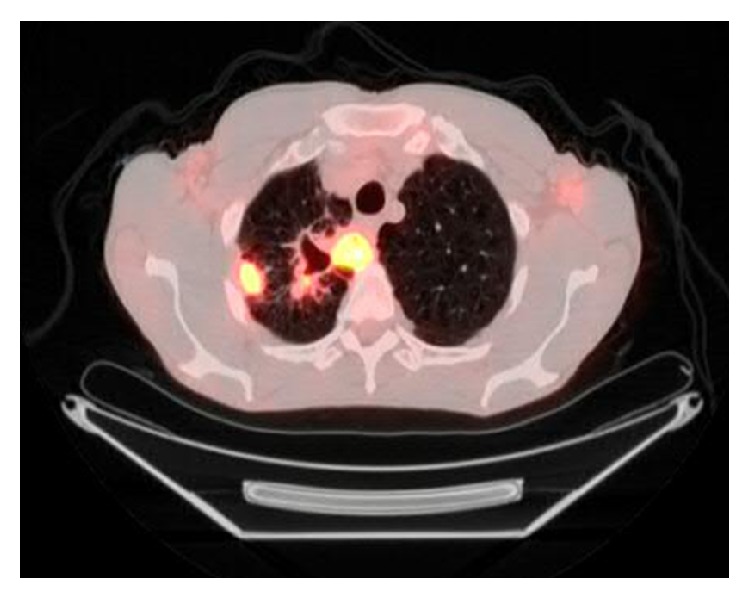
The ^18^F-FDG PET scan demonstrated hypermetabolic areas at the peripheral consolidation (SUV 7.4) and the apical cavitary mass (SUV 12.8) of the right upper lobe.

**Figure 4:**
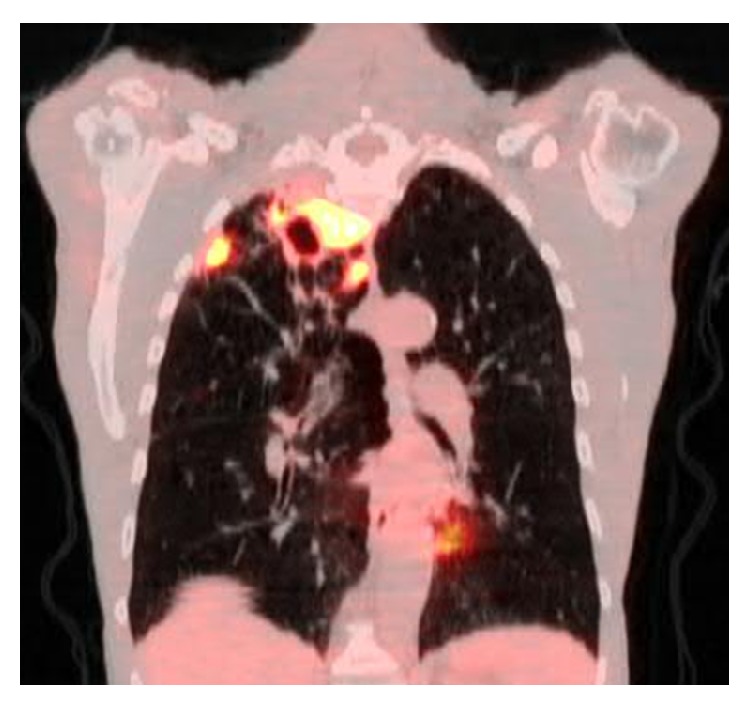
The ^18^F-FDG PET scan demonstrated hypermetabolic areas at the peripheral consolidation (SUV 7.4) and the apical cavitary mass (SUV 12.8) of the right upper lobe.

A 66-year-old male presented to his primary care physician with the right upper chest pain, progressive malaise, generalized weakness, loss of appetite, and a 10 lb weigh loss for 2 months. Past medical history was significant for rheumatoid arthritis, chronic pulmonary obstructive disease, and 60-pack-year of cigarette smoking. He had been treated for rheumatoid arthritis with weekly subcutaneous injection of etanercept for 10 months, and symptoms of rheumatoid arthritis were well-controlled. He denied cough, dyspnea, fever, chills, night sweat, or known tuberculosis exposure. Physical examination was unremarkable, and routine laboratory tests did not reveal significant abnormalities. A chest radiograph showed a right upper lobe density. Etanercept therapy was subsequently discontinued because of the concern of opportunistic infections secondary to etanercept. The tuberculin skin test (TST) was noted 2 mm prior to initiation of etanercept treatment. The patient underwent chest computed tomography (CT) which was reported a pleura-based consolidated mass (1.5 × 2 × 1 cm) on the lateral aspect of the right upper lobe and a thick-walled cavitary lesion (2.5 × 3 × 3.5 cm) on the superomedial side of the right lung apex, highly suggestive of a Pancoast tumor (Figures 1 and 2). There were background emphysematous pulmonary changes, without bronchiectasis, mediastinal, or hilar lymphadenopathy. It was followed by the ^18^F-FDG PET study, which demonstrated hypermetabolic uptake foci in these areas located within the right upper lobe (Figures 3 and 4). The standardized uptake values (SUVs) of the lesions were ranging from 7.4 to 12.8, consistent with pulmonary malignancy.

**Figure 5:**
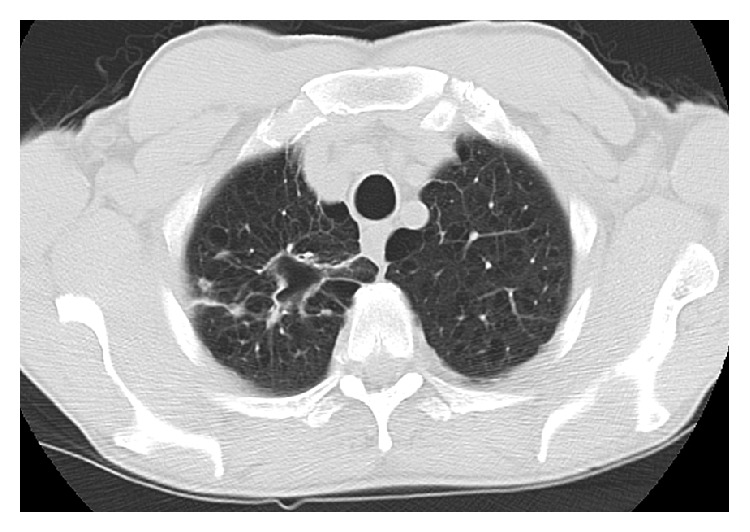
A follow-up CT chest after 6 weeks of antimycobacterial therapy revealed an interval resolution of the right pleura-based mass, and the right upper lobe cavity became thin-walled and much less prominent without new pulmonary lesions or lymphadenopathy.

**Figure 6:**
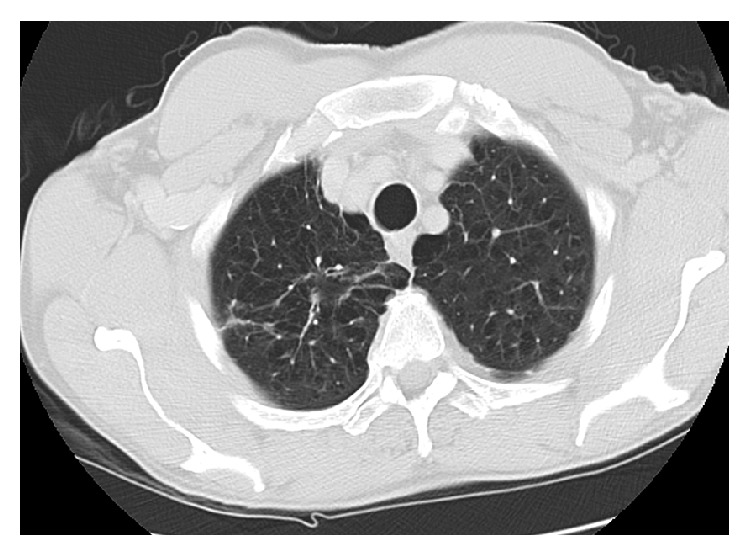
Right pleural mass was almost resolved, and right apical cavitary lesion had significantly improved after 6 months of the triple drug therapy.

**Figure 7:**
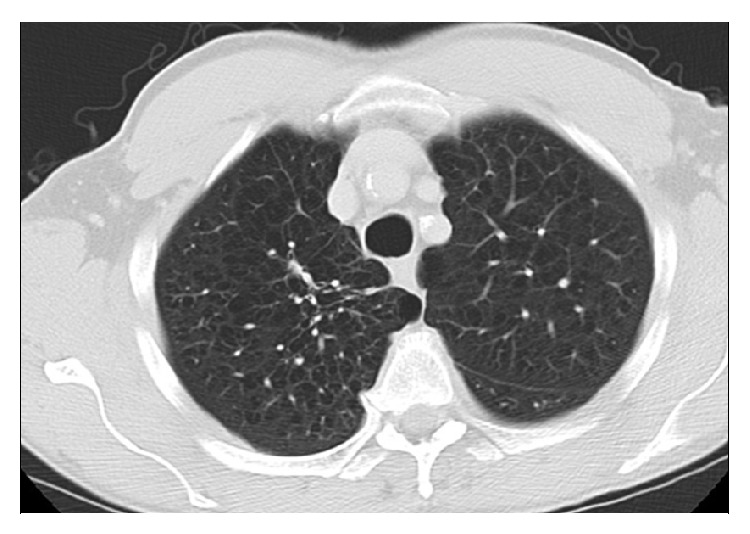
A follow-up CT chest at the end of the triple drug therapy showed a complete resolution of* Mycobacterium kansasii* pulmonary infection.

The bronchoscopy with bronchoalveolar lavage (BAL) was performed to establish the diagnosis of lung cancer. The bronchoscopy did not reveal endobronchial tumors. BAL cytology examination failed to demonstrate malignant cells. BAL Gomori methenamine-silver (GMS) stain and fungal culture was negative. The Ziehl-Neelsen acid-fast bacilli (AFB) smears of several BAL samples were positive. BAL* Mycobacterium tuberculosis* complex DNA probe and* Mycobacterium avium intracellulare* DNA probe were performed, and they were negative.* Mycobacterium kansasii* DNA probes on multiple BAL specimens reported positive, and a clinical diagnosis of pulmonary* M. kansasii* infection was then entertained. His HIV serology was negative. A triple drug regimen—isoniazid 300 mg/day, rifampin 600 mg/day, and ethambutol 1200 mg/day—was initiated while awaiting the final culture result. Chest pain and malaise were resolved in a week, and appetite returned in two weeks from the initiation of antimicrobial therapy.* M. kansasii* was eventually isolated from the culture after 6 weeks. The isolate was susceptible to rifampin, ethambutol, clarithromycin, rifabutin, ciprofloxacin, moxifloxacin, and amikacin. Serial CT chest imaging studies at the 6-week and the 6-month of antimycobacterial therapy revealed significant radiological improvement of pulmonary lesions (Figures 5 and 6). The patient continued to improve clinically, and a total resolution of pulmonary lesions was achieved (Figure 7) on completion of a 12-month course of triple drug therapy.

## 3. Discussion

Nontuberculous mycobacteria (NTM) are referred to mycobacteria species other than* Mycobacterium tuberculosis* complex (*M. tuberculosis*,* M. bovis*,* M. africanum*, and* M. microti*) and* M. leprae*. NTM have been found ubiquitously in environment (e.g., surface water, tap water, soil, domestic and wild animals, and dairy products) [5].

In the United States, infection with* M. kansasii* is known to be the second most common cause of NTM disease, after* M. avium intracellulare* infection [5, 6]. The incidence of pulmonary* M. kansasii* infection is geographically dominant in the southeastern and central United States [7, 8]. Unlike other NTM species, the tap water is the only known major environmental source for* M. kansasii* causing human disease [6]. The major risk factor for* M. kansasii* infection is chronic obstructive pulmonary disease, and other predisposing conditions include HIV infection, pneumoconiosis, immunosuppressants, alcoholism, and malignancy [6, 8].

The introduction of inhibitors of TNF-alpha for the treatment of autoimmune inflammatory diseases, such as rheumatoid arthritis, psoriasis, or inflammatory bowel disease, has a significant impact on the incidence of* Mycobacterium tuberculosis* (MTB) and NTM infections since TNF-alpha plays an important role in the human host defense mechanisms in containing mycobacterial infections [1]. Traditionally, it is considered a greater risk (5–10 times) of developing MTB than NTM infections in patients receiving TNF-alpha inhibitors [9]. However, another study reported NTM infections were more than MTB in patients treated with TNF-alpha blockers [2]. A more recent study analyzed the US Food and Drug Administration's MedWatch surveillance database to evaluate the incidence of NTM infections in patients using TNF-alpha inhibitors [3]. Among 105 confirmed cases of NTM,* M. avium intracellulare* was accounted for 52 cases (50%) whereas only 3 cases of* M. kansasii* (3%) were identified in the study. Notably, there is a differential risk stratification of MTB reactivation depending on the agent of TNF-alpha blockers [10]. Patients receiving infliximab and adalimumab generally have a higher risk of MTB reactivation than those with etanercept. However, there is no solid evidence to suggest the same principle of risk stratification applied to the NTM disease in patients on TNF-alpha inhibitors.

Clinical presentations of pulmonary* M. kansasii* infection are almost identical to MTB. Chest pain, cough, hemoptysis, fever, night sweats, malaise, and weight loss are observed in both infections. Radiographic features are very similar to reactivation of latent pulmonary MTB with upper lobe cavitary lesion [5, 6]. Treatment of* M. kansasii* infection is guided by susceptibility testing [11]. The initial susceptibility testing of* M. kansasii* isolate is recommended for rifampin only, and there is no additional sensitivity testing for rifabutin required for isolates that are susceptible to rifampin. For isolates resistant to rifampin, susceptibility testing for secondary drugs is recommended [5, 6, 11]. A panel of antimicrobial drug susceptibility is usually performed upfront to avoid a delay in optimal therapy. For rifampin-sensitive isolate, a 12-month course of three-drug combination therapy (from the first negative sputum culture) with isoniazid (5 mg/kg/day, maximum 300 mg/day), rifampin (10 mg/kg/day, maximum 600 mg/day), and ethambutol (15 mg/kg/day) is recommended. An alternative triple drug regimen for those who could not tolerate isoniazid is clarithromycin (500 mg twice daily), rifampin plus ethambutol. Rifabutin is preferred over rifampin in HIV patients to minimize drug-interaction with protease inhibitors [6, 11].

The thoracic ^18^F-FDG PET scan plays a major role in lung cancer staging, evaluation of treatment response after cancer therapy, and surveillance for cancer recurrence [12]. The ^18^F-FDG PET imaging is a nuclear medicine study modality, and its basic principle is tracing tissues with high glucose metabolism using fluoro-deoxy-glucose (FDG) as glucose analogue. Malignant tumor tissues are composed of actively dividing and proliferating cells and have significantly increased glycolytic mechanism, which could be detected by the ^18^F-FDG PET study. Therefore, it has increasingly been used to distinguish benign from malignant pulmonary nodules. The ^18^F-FDG-PET scan has high sensitivity of 96% and low specificity of 78% on detection of lung cancer [13]. The standardized uptake value (SUV) is calculated for the quantitative analysis of tumor uptake of FDG. Lesions with an SUV of above 2.5 are highly suggestive of malignancy [14]. Our patient's pulmonary lesions demonstrated markedly high SUVs ranging from 7.4 to 12.8, and they were therefore thought to be primary lung cancers. However, increased FDG uptake is not limited to malignant tissues but also inflammatory cells such as leukocytes, histiocytes, plasma cells, lymphocytes, and macrophages. Activated inflammatory cells have markedly high metabolic activity with increased glucose uptake and glycolysis process, and avid FDG uptake is undoubtedly the rule in inflammatory tissues. Thus, benign conditions with positive ^18^F-FDG-PET scan findings have been reported in an array of infections, inflammatory, or granulomatous diseases (Table 1), but the list is not exhaustive [4, 15–18].

Our patient's clinical manifestations and chest radiographic appearance provided a broad range of differential diagnoses, including infections (bacteria, mycobacteria, or fungi) and malignancy. In our patient, the clinical decision making was instead influenced by the chest CT report. A strongly positive ^18^F-FDG PET scan further stirred up unwarranted anxiety to the treating physician and patient alike.

To the best of our knowledge, our case is the first report of positive thoracic ^18^F-FDG PET scan of pulmonary* M. kansasii* infection in a patient receiving a TNF-alpha inhibitor. There were two other published case reports of nonpulmonary* M. kansasii* infections (skin lesions and olecranon bursitis) in patients treated with anti-TNF-alpha agents (Table 2) [19, 20]. A case of ^18^F-FDG PET-positive solitary pulmonary nodule from* M. kansasii* infection in an immunocompetent host was reported in the literature [18].

In conclusion, our case highlights an increased risk of* M. kansasii* lung disease in patients receiving etanercept, and it is therefore important to rule out infectious etiologies in these patients presenting with pulmonary pathology. Positive ^18^F-FDG PET results should be interpreted with great caution in differentiating infections from malignant conditions. Microbiologic and histopathologic examination remains the gold standard to differentiate these two groups. Our case emphasizes the limitations and pitfalls of ^18^F-FDG PET imaging study, such as low specificity and high cost, and its application should be judiciously utilized in cases of histologically proven malignancy.

## Figures and Tables

**Table 1:** A nonexhaustive list of reported nonmalignant conditions of positive ^18^F-FDG PET scan [4, 15–18].

Infectious causes	Noninfectious causes
MTB and MTB lymphadenopathy	Pulmonary granuloma/granulation tissue
NTM infections (e.g., *M. avium intracellulare*, *M. kansasii*)	Hypersensitivity pneumonitis
Pyogenic abscesses	Radiation pneumonitis
Bacterial pneumonia	Thyroiditis
Osteomyelitis	Sarcoidosis
Aspergillosis	Pneumoconiosis
Histoplasmosis	Thymus hyperplasia
Cryptococcosis	Paget's disease of bone
*Pneumocystis jirovecii* pneumonia	Chronic pancreatitis
Pulmonary paragonimiasis	Retroperitoneal fibrosis

MTB—*Mycobacterium tuberculosis*; NTM—nontuberculous mycobacteria.

**Table 2:** A selected list of published case reports of *M. kansasii* infection in patients on anti-TNF-alpha drugs [19, 20].

Reference	Age (years)/gender	Underlying disease	Site of infection	Type of TNF-alpha inhibitor	Antimicrobial therapy	Outcome
[19]	48/female	Sarcoidosis	Confluent vesicles on the dorsum of the left foot	Etanercept, followed by infliximab	R, I, E x 9 months	Resolved
[20]	53/female	Behcet's disease	Bilateral olecranon bursitis	Infliximab	R, ∗Cl/Ci, E x 19 months; Bursectomy	Resolved
Index case	66/male	Rheumatoid arthritis	Lung	Etanercept	R, I, E x 12 months	Resolved

R: rifampin; I: isoniazid; and E: ethambutol.

∗Cl/Ci: clarithromycin (Cl) was changed to ciprofloxacin (Ci) after 2 weeks due to persistent nausea; ciprofloxacin was stopped after 2 months of therapy because of Achilles tendinitis. The patient completed rifampin and ethambutol for 19 months.
